# Developmental Neurotoxicity of Methamidophos in the Embryo-Larval Stages of Zebrafish

**DOI:** 10.3390/ijerph14010023

**Published:** 2016-12-28

**Authors:** Xiaowei He, Jiawei Gao, Tianyu Dong, Minjian Chen, Kun Zhou, Chunxin Chang, Jia Luo, Chao Wang, Shoulin Wang, Daozhen Chen, Zuomin Zhou, Ying Tian, Yankai Xia, Xinru Wang

**Affiliations:** 1 State Key Laboratory of Reproductive Medicine, Institute of Toxicology, School of Public Health, Nanjing Medical University, Nanjing 211166, China; hexiaoweicy@163.com (X.H.); 13057522761@163.com (J.G.); tydong@hotmail.com (T.D.); zk@njmu.edu.cn (K.Z.); changchunxin1990@163.com (C.C.); xixiazure@gmail.com (J.L.); chaowang_sph_njmu@163.com (C.W.); wangshl@njmu.edu.cn (S.W.); 2 Key Laboratory of Modern Toxicology of Ministry of Education, School of Public Health, Nanjing Medical University, Nanjing 211166, China; 3 Wuxi Maternal and Child Health Hospital, Nanjing Medical University, Wuxi 214002, China; chendaozhen@163.com; 4 State Key Laboratory of Reproductive Medicine, Nanjing Medical University, Nanjing 211166, China; zhouzm@njmu.edu.cn; 5 MOE and Shanghai Key Laboratory of Children’s Environmental Health, Xinhua Hospital, School of Medicine, Shanghai Jiao Tong University, Shanghai 200092, China; tianmiejp@shsmu.edu.cn; 6 Department of Environmental Health, School of Public Health, Shanghai Jiao Tong University, Shanghai 200025, China

**Keywords:** methamidophos, developmental neurotoxicity, zebrafish, toxicology

## Abstract

Methamidophos is a representative organophosphate insecticide. The knowledge of its developmental neurotoxicity is limited, especially for zebrafish in the early stages of their life. Four hour post-fertilization (hpf) zebrafish embryos were exposed to several environmentally relevant concentrations of methamidophos (0, 25, and 500 μg/L) for up to 72 hpf. Locomotor behavior was then studied in the zebrafish larvae at this timepoint. Acridine orange (AO) staining was carried out in the zebrafish larvae, and the mRNA levels of genes associated with neural development (*mbp* and *syn2a*) were analyzed by reverse transcription-polymerase chain reaction (RT-PCR). The number of escape responders for mechanical stimulation was significantly decreased in exposed groups. AO staining showed noticeable signs of apoptosis mainly in the brain. In addition, the mRNA levels of *mbp* and *syn2a* were both significantly down-regulated in exposed groups. Our study provides the first evidence that methamidophos exposure can cause developmental neurotoxicity in the early stages of zebrafish life, which may be caused by the effect of methamidophos on neurodevelopmental genes and the activation of cell apoptosis in the brain.

## 1. Introduction

Globally, organophosphate (OP) pesticides have been widely used in agriculture, forestry and gardening, which account for almost 40% of total pesticide sales by volume [1]. A Brazilian study demonstrated that over 90% of the farmers use pesticide products containing methamidophos as active ingredient and nearly 60% of them present typical OP intoxication symptoms [2]. Methamidophos, a representative OP pesticide, has been used extensively in agriculture to limit pest damages to cultivated plants [3]. In Latin America, methamidophos is now the second most used active ingredient of pesticide products [4]. Methamidophos is persistently contaminating crops and used as a suicidal agent in many countries, especially in developing countries [5]. In China, methamidophos residues were still detectable in vegetables in marketplaces in 2015 [6]. A great number of studies have reported that the residual levels of methamidophos among various fruits and vegetables in developing countries such as Pakistan and Brazil exceeded the maximum residue limits (MRLs) established by the World Health Organization (WHO) and the European Union (EU) legislation in recent years [7,8]. Thus, methamidophos toxicity has been an interesting topic in public health studies in recent years [1,9,10,11]. Notably, Rodriguez et al. [12] reported that long-term exposure to methamidophos for pregnant women ranged from 3 to 1003 h, and for children ranged from 6 to 1964 h. Therefore, a great attention should be paid into the potential toxicity of methamidophos, especially during early life of humans.

During the past decade, many studies using models based on adult rodents exposed to methamidophos have noted that it affects cardiovascular reflexes, alters spermatogenesis and induces delayed neuropathy [9,13,14]. In addition, many studies have demonstrated that methamidophos could lead to specific neuronal damages, and might inflict delayed neuropathy in adult rodents [15,16]. Notably, methamidophos has both hydrophilic and hydrophobic domains [17], and it can pass through the blood-brain barrier and placenta, which has also been found in amniotic fluid [18]. Vidair reported that OP_S_ were more neurotoxic in young rats compared with adults [19]. In addition, fetuses, infants and young children may be more susceptible to the potential neurotoxic effects of pesticides, because their brains are developing rapidly [20]. However, the developmental neurotoxicity of methamidophos in the early life stage is still largely unknown. Therefore, further toxicological research of methamidophos is required. Zebrafish and its embryos, which have many advantages, such as a short lifecycle, high fecundity and transparency during early life stages, are widely used as models for studies of developmental toxicity [21]. These advantages make it easy to evaluate morphological endpoints and observe developmental processes [22,23]. Furthermore, many characteristics of zebrafish are similar to humans, such as development, metabolism and compoumd-induced organ/tissue reaction [24]. This may be due to the fact many molecular pathways are evolutionarily conserved between humans and zebrafish [25,26]. Thus, zebrafish represent a reasonable and effective model for evaluating the developmental neurotoxicity of chemicals in aquatic environments [27]. However, few studies have studied the developmental neurotoxicity of methamidophos in the early embryonic stages of zebrafish.

This study was therefore designed to study methamidophos neurotoxicity and related mechanism in the early stages of zebrafish. Two environmentally relevant concentrations of methamidophos (25 and 500 μg/L) were selected, which were according to the concentration in water environment and the level in part of the human samples, respectively [28,29]. The analysis of locomotor behavior and apoptosis was used to assess the impairment of nervous system. Reverse transcription-polymerase chain reaction (RT-PCR) was employed to compare the mRNA levels of genes associated with neural development between the control and exposed groups. All of the information firstly acquired in this study will be helpful to understand the developmental neurotoxicity induced by methamidophos in aquatic systems.

## 2. Materials and Methods

### 2.1. Chemicals and Reagents

Methamidophos (O,S-dimethyl phosphoramidothioate; CAS Number: 10265-92-6; purity: 99.70%) was purchased from Dr. Ehrenstorfer (Augsburg, Germany). Acridine orange (AO; CAT. No. 235474) staining reagent, Hanks’ Balanced Salt Solution (HBSS; CAT. No. H6648) and tricaine (MS-222; CAT. No. A5040) were purchased from Sigma-Aldrich (St. Louis, MO, USA). Total RNA was extracted by TRIzol (Invitrogen, Carlsbad, CA, USA). Reverse transcription and SYBR-green RT-PCR reagents (CAT. No. RR036A; RR420A) were obtained from TaKaRa (Tokyo, Japan). All of the other chemicals required in this study were of analytical grade.

### 2.2. Zebrafish Husbandry and Collection of Embryos

AB strain zebrafish (*Danio rerio*), acquired from the model animal center of Nanjing University were kept in a recirculating system at 28.5 °C, with a 14 h light/10 h dark cycle 7 days per week [30]. Fish system water was aerated and measured daily to maintain dissolved oxygen concentration at 7.5–8 mg/L and pH at 7.0–7.6. The fish were fed with live brine shrimp (*Artemia nauplii*, Tianjin Ocean Pal Carol Biotech Co., Tianjin, China) twice a day. Male and female adult fish with a preferable ratio of 2:1 were transferred in pairs overnight in a spawning aquarium, and the spawning finished at the first 30 min of the light cycle on the next morning. Fertilized eggs were collected, washed with embryo medium (0.137 M NaCl, 5.4 mM KCl, 0.25 mM Na_2_HPO_4_, 0.44 mM KH_2_PO_4_, 1.3 mM CaCl_2_, 1.0 mM MgSO_4_ and 4.2 mM NaHCO_3_) and incubated in Petri dishes at 28 ± 1 °C [31] until methamidophos exposure experiments. All experimental procedures involving animals were conducted in accordance with the guide for the Care and Use of Laboratory Animals of the National Institutes of Health (NIH, Bethesda, MD, USA) and were approved by the Committee on the Ethics of Animal Experiments of Nanjing Medical University (Permit number: 2014092). All efforts were made to minimize animal suffering and to reduce the number of animals used.

### 2.3. Methamidophos Treatment

The methamidophos administration was conducted according to a previous study [32]. In brief, test solutions with methamidophos were prepared freshly using embryo medium before exposure experiments. The nominal concentrations of the test solutions were 0 (control), 25 μg/L (0.177 μM), 500 μg/L (3.543 μM). Zebrafish embryos of 4 h post-fertilization (hpf) were transferred to the solutions of series of concentrations in a 96-well plate with one larvae per well, and the plate was incubated at 28 ± 1 °C under the same light cycle as the adults throughout the 72 h exposure period. The exposure solutions were changed daily to ensure the nominal concentrations of methamidophos and water quality. Three replicates were set up for the control and each treatment.

### 2.4. The Observations of Morphological Development

The observations of zebrafish morphological development were performed according to a previous report [33]. Briefly, the observations were conducted directly in the 96-well plate using an inverted dissecting microscope (Leica Microsystems, Wetzlar, Germany). The embryos and larvae were evaluated for morphological change at 24 and 72 hpf, respectively.

### 2.5. Locomotor Behavior Observation

Locomotor behavior observation was conducted according to a previous report [34]. The observations of locomotor behavior were performed directly in the 60-mm glass Petri dishes using an inverted dissecting microscope. The 72-hpf-larvae were evaluated for their response to a mechanical stimulus (touch). Each larvae was observed for a response to three stimuli which gently touched on the head using a probe [35]. To prevent any pre-stimulus modulation of the response, we waited for 5 s between two stimuli [36]. Larvae that swam away after all the three repeated stimuli were scored as responders while all others were scored as non-responders [37]. The number of escape responders for mechanical stimulation was ultimately recorded for each dose group. Locomotor behavior was observed in three replicates (20 larvae) per treatment [38].

### 2.6. AO Staining

Embryo cell apoptosis was assessed using AO, a widely used nucleic acid-selective metachromatic stain [39,40]. AO staining was conducted according to a previous report [41]. At 72 hpf, after exposure to a series of methamidophos concentrations (0, 25, and 500 μg/L), 10 living larvae from each replicate were washed twice in Hank’s Balanced Salt Solution (HBSS), and then transferred into 5 mg/L of AO staining dissolved in HBSS in darkness for 30 min at room temperature. The larvae were then washed twice in HBSS thoroughly. Before examination, the larvae were anesthetized with 0.03% MS-222 for 3 min. Stained larvae were photographed under a fluorescence microscope (Nikon, Tokyo, Japan). The apoptotic cells appear overt bright spots [42]. In order to determine the fluorescence intensity in the brain regions, florescence intensities on the staining of the region of interest (ROI) were measured on a total of 30 larvae with software Image J (National Institutes of Health, Bethesda, MD, USA) [43]. Different brain regions were evaluated on account of surrounding landmarks [44].

### 2.7. RNA Isolation and Real-Time PCR Assays

At 72 hpf, after exposure to a series of methamidophos, total RNA was extracted from 20 living zebrafish larvae using Trizol reagent (Invitrogen, Carlsbad, CA, USA) according to the manufacturer’s instructions, and the concentration of total RNA was measured with NanoDrop 2000 (Thermo Fisher Scientific, Wilmington, DE, USA). Synthesis of first-strand cDNA was performed by reverse transcription of 1 mg total RNA with M-MLV RTase (TaKaRa, Tokyo, Japan). The mRNA levels of *myelin basic protein* (*mbp*) and *synapsin IIa* (*syn2a*) were analyzed using SYBR Green PCR Master Mix reagent kits (TaKaRa, Tokyo, Japan). All RT-PCR were performed on an ABI 7900 Fast Real-Time System (Perkin-Elmer Applied Biosystems, Foster City, CA, USA). The thermal cycle was set at 95 °C for 2 min, followed by 40 cycles at 95 °C for 15 s, 60 °C for 15 s, and 72 °C for 1 min. The oligonucleotide primers utilized in this study were synthesized by Invitrogen (Shanghai, China) and the primer sequences are shown in Table 1. The 2^−ΔΔCt^ method was used to calculate the relative mRNA expression levels of genes normalized by the *β-actin* gene [45]. Each sample was run in triplicate [46].

### 2.8. Statistical Analysis

The differences between three groups were evaluated by non-parametric test (Kruskal-Waillis with multiple comparison tests) to identify significant differences. All statistical analysis was conducted using Statistical Analysis System (version 9.4, SAS Institute, Cary, NC, USA) and the criterion of significance was set at *p* < 0.05. All values were expressed as mean ± standard error of the mean (SEM).

## 3. Results

### 3.1. Morphological Development Observation

**Figure 1:**
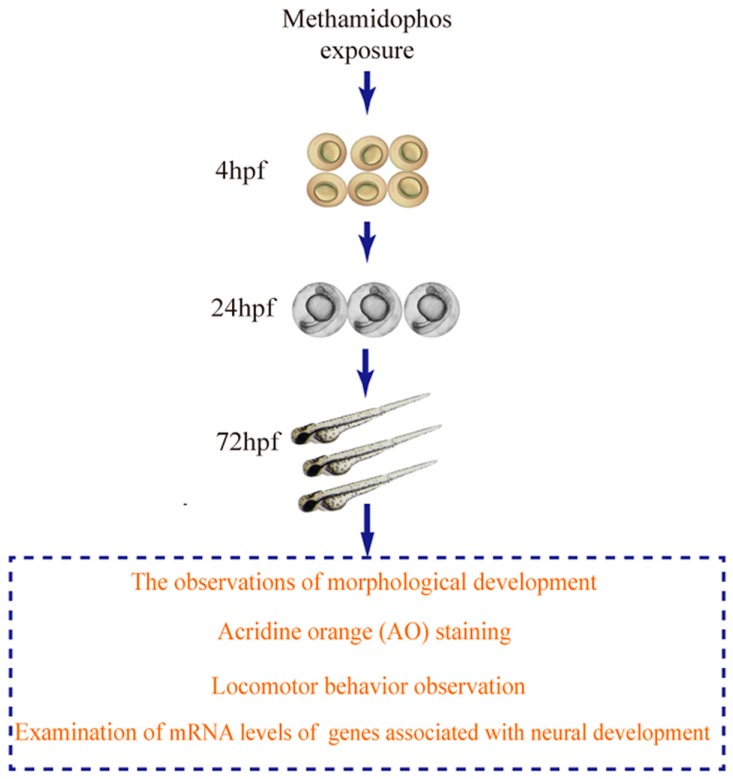
Experimental work flow of this study.

Our experimental flow diagram is presented in Figure 1.

**Figure 2:**
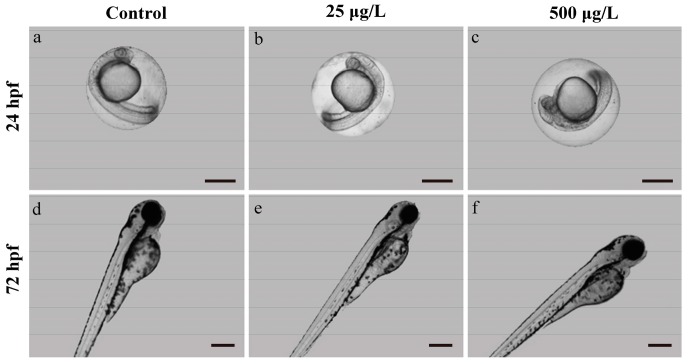
Representative morphological photographs of zebrafish exposed to methamidophos at 24 and 72 hpf. Scale bars: 500 µm (**a**–**c**) and 250 µm (**d**–**f**).

In the morphology development analysis, we found the methamidophos-exposed zebrafish larvae did not show obvious delay of morphological development in both 24 hpf and 72 hpf (Figure 2).

### 3.2. Abnormal Locomotor Behavior of Methamidophos-Exposed Zebrafish Larvae

**Figure 3:**
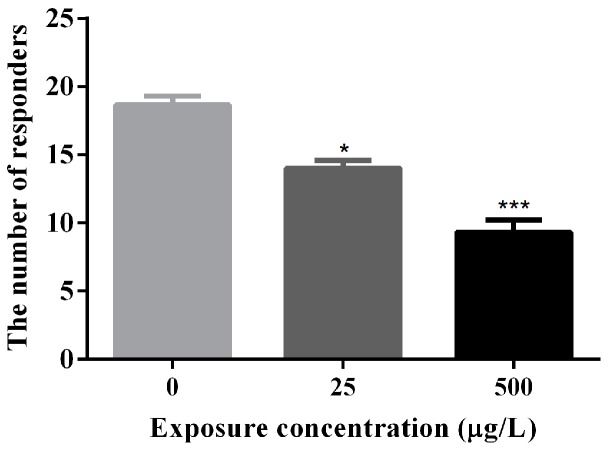
Locomotor behavior of methamidophos-exposed zebrafish larvae at 72 hpf. * *p* < 0.05; *** *p* < 0.001.

The number of escape responders for mechanical stimulation was summarized in Figure 3 and Table S1. The response to a mechanical stimulus can be used as a measurement of sensorimotor integration [47]. Some of the larvae in exposure groups did not have obvious response to a mechanical stimulus. The number of escape responders for mechanical stimulation was reduced to half of the control group at high-dose group based on normal morphology.

### 3.3. Apoptosis in Zebrafish Larvae Induced by Methamidophos Exposure

**Figure 4:**
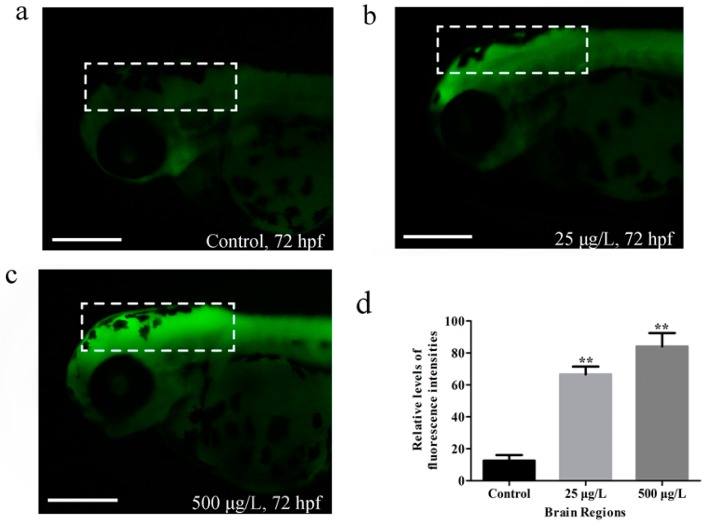
Apoptosis in the brain of zebrafish larvae induced by methamidophos exposure detected with AO staining at 72 hpf. Scale bars: 250 µm (**a**–**c**). Relative levels of fluorescence intensity in the brain regions (**d**). ** *p* < 0.01.

We next examined the zebrafish larvae at 72 hpf using AO staining, which could reveal the developmental neurotoxic chemical-induced apoptosis in nervous system. Apoptotic cells of the living larvae in each group were revealed by AO staining. No obvious apoptotic cells were observed in zebrafish larvae of the control group, whereas notable signs of apoptosis appeared in zebrafish larvae of each exposure group, mainly around the brain with bright green fluorescent spots (Figure 4a–c). The fluorescence intensities of the brain regions were both significantly higher in exposed groups than in the control group (Figure 4d) (** *p* < 0.01, respectively for the two exposure groups).

### 3.4. Effects of Methamidophos on the mRNA Levels of Genes Associated with Neural Development

**Figure 5:**
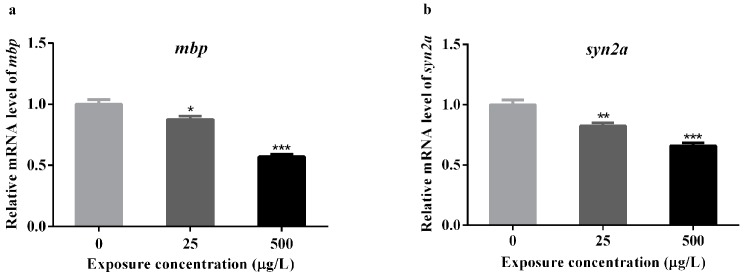
The mRNA levels of two genes associated with neural development in the zebrafish larvae exposed to several concentrations of methamidophos at 72 hpf. The expression levels of the *mbp* (**a**) and *syn2a* (**b**) genes were both significantly down-regulated in the 25 and 500 μg/L exposure groups, compared with the control group. * *p* < 0.05; ** *p* < 0.01; *** *p* < 0.001.

Based on the results of abnormal locomotor behavior and brain apoptosis induced by methamidophos, the expression alterations of *mbp*, and *syn2a* genes in zebrafish were detected after methamidophos exposure at 72 hpf (Table S2). The gene of *mbp* which expresses in oligodendrocytes of myelin sheath, typically serves as a biomarker of myelination of axons in the developing central nervous system (CNS) of zebrafish and human [48,49]. The gene of *syn2a* is a biomarker of synapse formation in mammals, playing an important role in synaptogenesis and neurotransmitter release [50]. The *mbp* expression was significantly down-regulated in larvae from the 25 and 500 μg/L exposure groups, compared with the control (* *p* < 0.05 and *** *p* < 0.001, respectively for the two exposure groups) (Figure 5a). Similarly, there was also an obvious decrease in the expression of *syn2a* (** *p* < 0.01 and *** *p* < 0.001, respectively for the two exposure groups) (Figure 5b). Notably, the expression of *mbp* in 500 μg/L exposure group was almost reduced to half of the control group, which was 0.57 fold change relative to control.

## 4. Discussion

**Figure 6:**
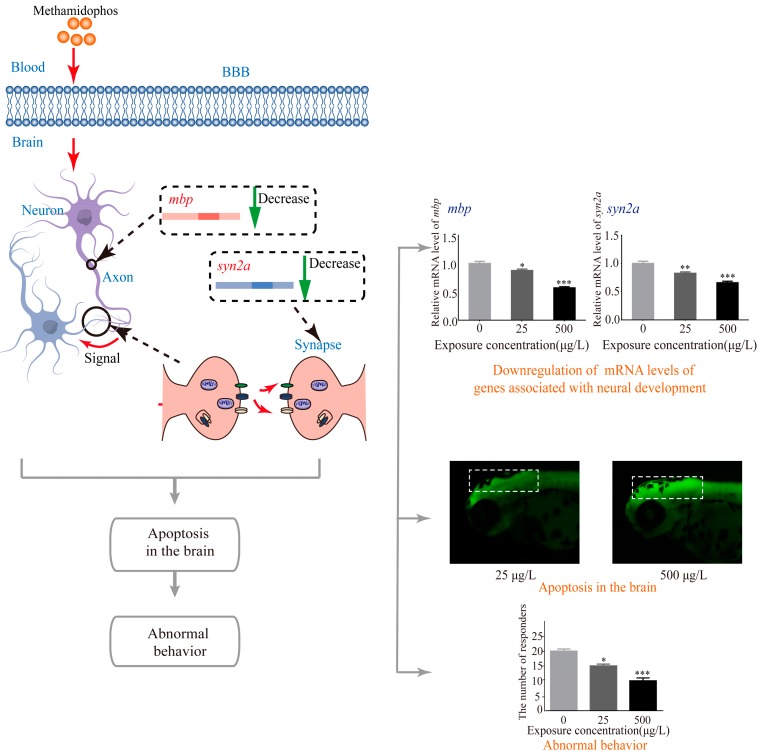
The developmental neurotoxicity of methamidophos in embryo-larval stages of zebrafish. * *p* < 0.05; ** *p* < 0.01; *** *p* < 0.001.

As far as we know, this is the first study to report the developmental neurotoxicity and possible mechanisms in terms of exposure to methamidophos in the embryo-larval stages of zebrafish. By the combination of studies regarding larval locomotor behavior, AO staining and transcript expressions, we demonstrated that exposure to environmentally relevant methamidophos in zebrafish embryo and larvae could cause developmental neurotoxicity (Figure 6). 

Methamidophos is water soluble and has a moderate potential for runoff into surface waters. One of the concentrations of methamidophos exposure (25 μg/L) is close to environmentally relevant concentrations of methamidophos in the water environment of some regions, such as Philippines, India and other countries [28]. Besides, another concentration (500 μg/L) is near to the level of methamidophos in part of the human samples [29,51,52], which indicates that people in some countries are exposed to methamidophos in dangerous levels. Besides, many studies have reported that methamidophos concentration in fresh fruits and vegetables were higher than the FAO/WHO permissible limits residual levels [7,53]. Thus, attention should be paid to the developmental neurotoxicity of methamidophos.

Screening for neurotoxicity can be assessed by motor, sensory, autonomic, and integrative neurological functions. Behavior represents the sum of activities controlled by the nervous system, which can evaluate the consequences of disruption of neuronal communications [54]. Behavior test is widely used in neurotoxicity testing of pharmaceutical and environmental chemicals [55,56]. Thus, locomotor behavior of zebrafish larvae was observed to reflect potential neurotoxicity of methamidophos in our study. It is reported that hatched-stage larvae can show a mature escape reflex in response to tactile stimulation, and this behavior can serve as a simple assay for sensorimotor integration [37]. However, the response to mechanical stimuli of larvae in exposure groups was decreased in our results. This loss of touch-induced escape response indicated probably sensorimotor damage in zebrafish and developmental neurotoxicity of methamidophos, which is supported by previous studies on developmental neurotoxicity of environmental chemicals in zebrafish [57,58].

Apoptosis, also be referred to as programmed cell death [59], is an important regulator of growth and development. In this study, apoptotic cells were specially observed in the brain of the zebrafish larvae by AO staining (Figure 4), suggesting that the developing brain was very likely to be an important target for methamidophos in zebrafish. An increase of apoptosis in the brain induced by methamidophos might contribute to the abnormal neurodevelopment, and might explain the abnormal behavior of zebrafish larvae induced by methamidophos. Methamidophos has both hydrophilic and hydrophobic domains [17], therefore, it is reasonable to expect that methamidophos might go through the blood brain barrier (BBB) by diffusion. Besides, an active transport mechanism might also be involved in the penetration process [60].

It is widely demonstrated that the structure, synthesis and gene expression of myelin sheath are highly conserved between zebrafish and mammals [61,62]. The expression level of *mbp* gene was down-regulated in our study, suggesting that exposure to methamidophos might affect the function of oligodendrocytes, and further affect the formation of myelin sheath. Similarly, a recent study shows that exposure to chlorpyrifos, which is another widely used OP, resulted in reduced *mbp* in the early life stage of zebrafish [63]. Recently, a study pointed out that *syn2a* was increasingly expressed during the process of nervous system differentiation, implying that synapsins is crucial for neuronal differentiation and synaptogenesis in the early developmental stages of CNS [64]. Therefore, the downregulation of *syn2a* observed in this study may affect neuronal differentiation and synaptogenesis, and ultimately lead to neurobehavioral impairments.

The developmental neurotoxicity of methamidophos has been studied in rodents. Moser found that methamidophos (4 and 8 mg/kg for post-natal day 17) could alter neurobehavior in young rats [65]. Lima et al. demonstrated that methamidophos (1 mg/kg/day, from post-natal days 3 to 9) was deleterious to the developing brain and could elicit behavioral alterations in mice [66]. Moreover, Castro et al. evaluated the influence of oral exposure to methamidophos during gestational organogenesis of rats (1 mg/kg/day, from gestational days 6 to 15), and they found methamidophos showed suggestive effects on behavioral development [67]. These studies indicated developmental neurotoxicity of methamidophos in rodents, supporting our findings in fish. Our study firstly explored the developmental neurotoxicity of methamidophos using zebrafish model, which provided the evidence of developmental neurotoxicity of methamidophos in different species.

## 5. Conclusions

This is the first study to reveal the induction of developmental neurotoxicity in zebrafish by environmentally relevant concentration of methamidophos. Methamidophos decreases the number of escape responders for mechanical stimulation through the action of methamidophos on neurodevelopmental genes and the activation of cell apoptosis in the brain in embryo-larval stages of zebrafish. Our study provides novel insights into methamidophos induced developmental neurotoxicity and its underlying mechanism, and highlights the importance on studying the developmental neurotoxicity of methamidophos in other aquatic species and humans.

## Figures and Tables

**Table 1:** Sequences of primers for the genes tested.

Target Gene	GenBank Accession No.	Primer Sequences
*mbp*	AY860977	Forward: 5’-AATCAGCAGGTTCTTCGGAGGAGA-3’ Reverse: 5’-AAGAAATGCACGACAGGGTTGACG-3’
*syn2a*	NM_001002597	Forward: 5’-GTGACCATGCCAGCATTTC-3’
		Reverse: 5’-TGGTTCTCCACTTTCACCTT-3’
*β-actin*	AF025305	Forward: 5’-ACAGGGAAAAGATGACACAGATCA-3’ Reverse: 5’-CAGCCTGGATGGCAACGTA-3’

## Supplementary Materials

The following are available online at www.mdpi.com/1660-4601/14/1/23/s1, Table S1: Behavior observation of zebrafish larvae exposed to methamidophos at 72 hpf, Table S2: The mRNA levels of *mbp* and *syn2a* in zebrafish larvae exposed to methamidophos at 72 hpf.

## References

[1] Ibhazehiebo K., Iyawe V.I., Koibuchi N. (2013). Effect of methamidophos on cerebellar neuronal cells. Niger. J. Physiol. Sci..

[2] Recena M.C., Caldas E.D., Pires D.X., Pontes E.R. (2006). Pesticides exposure in Culturama, Brazil—Knowledge, attitudes, and practices. Environ. Res..

[3] Gubert P., Avila D.S., Bridi J.C., Saurin S., Lugokenski T.H., Villarinho J.G., Fachinetto R., Pereira M.E., Ferreira J., da Rocha J.B. (2011). Low concentrations of methamidophos do not alter ache activity but modulate neurotransmitters uptake in hippocampus and striatum in vitro. Life Sci..

[4] Quinteros E., Ribo A., Mejia R., Lopez A., Belteton W., Comandari A., Orantes C.M., Pleites E.B., Hernandez C.E., Lopez D.L. (2016). Heavy metals and pesticide exposure from agricultural activities and former agrochemical factory in a Salvadoran rural community. Environ. Sci. Pollut. Res. Int..

[5] Rojas-Garcia A.E., Medina-Diaz I.M., Robledo-Marenco M.L., Barron-Vivanco B.S., Giron-Perez M.I., Velazquez-Fernandez J.B., Gonzalez-Arias C.A., Albores-Medina A., Quintanilla-Vega B., Ostrosky-Wegman P. (2011). Hematological, biochemical effects, and self-reported symptoms in pesticide retailers. J. Occup. Environ. Med..

[6] Yu R., Liu J.S., Wang Q.C., Liu Q., Wang Y. (2015). Contamination of organophosphorus pesticides residue in fresh vegetables and related health risk assessment in Changchun, China. Huan Jing Ke Xue.

[7] Syed J.H., Alamdar A., Mohammad A., Ahad K., Shabir Z., Ahmed H., Ali S.M., Sani S.G., Bokhari H., Gallagher K.D. (2014). Pesticide residues in fruits and vegetables from Pakistan: A review of the occurrence and associated human health risks. Environ. Sci. Pollut. Res. Int..

[8] Andrade G.C., Monteiro S.H., Francisco J.G., Figueiredo L.A., Botelho R.G., Tornisielo V.L. (2015). Liquid chromatography-electrospray ionization tandem mass spectrometry and dynamic multiple reaction monitoring method for determining multiple pesticide residues in tomato. Food Chem..

[9] Uriostegui-Acosta M., Hernandez-Ochoa I., Sanchez-Gutierrez M., Pina-Guzman B., Rafael-Vazquez L., Solis-Heredia M.J., Martinez-Aguilar G., Quintanilla-Vega B. (2014). Methamidophos alters sperm function and DNA at different stages of spermatogenesis in mice. Toxicol. Appl. Pharmacol..

[10] Peng H.F., Bao X.D., Zhang Y., Huang L., Huang H.Q. (2015). Identification of differentially expressed proteins of brain tissue in response to methamidophos in flounder (*Paralichthys olivaceus*). Fish Shellfish Immunol..

[11] Ramirez-Vargas M.A., Huerta-Beristain G., Guzman-Guzman I.P., Alarcon-Romero L.D., Flores-Alfaro E., Rojas-Garcia A.E., Moreno-Godinez M.E. (2015). Methamidophos induces cytotoxicity and oxidative stress in human peripheral blood mononuclear cells. Environ. Toxicol..

[12] Rodriguez T., van Wendel de Joode B., Lindh C.H., Rojas M., Lundberg I., Wesseling C. (2012). Assessment of long-term and recent pesticide exposure among rural school children in Nicaragua. Occup. Environ. Med..

[13] Maretto G.X., do Nascimento C.P., Passamani L.M., Schenberg L.C., de Andrade T.U., Figueiredo S.G., Mauad H., Sampaio K.N. (2012). Acute exposure to the insecticide O,S-dimethyl phosphoramidothioate (methamidophos) leads to impairment of cardiovascular reflexes in rats. Ecotoxicol. Environ. Saf..

[14] Emerick G.L., DeOliveira G.H., dos Santos A.C., Ehrich M. (2012). Mechanisms for consideration for intervention in the development of organophosphorus-induced delayed neuropathy. Chem. Biol. Interact..

[15] Noriega-Ortega B.R., Armienta-Aldana E., Cervantes-Pompa J.A., Hernandez-Ruiz E., Chaparro-Huerta V., Bravo-Cuellar A., Beas-Zarate C. (2011). Gaba and dopamine release from different brain regions in mice with chronic exposure to organophosphate methamidophos. J. Toxicol. Pathol..

[16] Emerick G.L., Peccinini R.G., de Oliveira G.H. (2010). Organophosphorus-induced delayed neuropathy: A simple and efficient therapeutic strategy. Toxicol. Lett..

[17] Singh A.K., White T., Spassova D., Jiang Y. (1998). Physicochemical, molecular-orbital and electronic properties of acephate and methamidophos. Pharmacol. Toxicol. Endocrinol..

[18] Bradman A., Barr D.B., Claus Henn B.G., Drumheller T., Curry C., Eskenazi B. (2003). Measurement of pesticides and other toxicants in amniotic fluid as a potential biomarker of prenatal exposure: A validation study. Environ. Health Perspect..

[19] Vidair C.A. (2004). Age dependence of organophosphate and carbamate neurotoxicity in the postnatal rat: Extrapolation to the human. Toxicol. Appl. Pharmacol..

[20] Rice D., Barone S. (2000). Critical periods of vulnerability for the developing nervous system: Evidence from humans and animal models. Environ. Health Perspect..

[21] Shi X., Du Y., Lam P.K., Wu R.S., Zhou B. (2008). Developmental toxicity and alteration of gene expression in zebrafish embryos exposed to PFOS. Toxicol. Appl. Pharmacol..

[22] Yang L., Ho N.Y., Alshut R., Legradi J., Weiss C., Reischl M., Mikut R., Liebel U., Muller F., Strahle U. (2009). Zebrafish embryos as models for embryotoxic and teratological effects of chemicals. Reprod. Toxicol..

[23] Zhou S., Dong Q., Li S., Guo J., Wang X., Zhu G. (2009). Developmental toxicity of cartap on zebrafish embryos. Aquat. Toxicol..

[24] Goldsmith J.R., Jobin C. (2012). Think small: Zebrafish as a model system of human pathology. J. Biomed. Biotechnol..

[25] Canestro C., Yokoi H., Postlethwait J.H. (2007). Evolutionary developmental biology and genomics. Nat. Rev. Genet..

[26] Lieschke G.J., Currie P.D. (2007). Animal models of human disease: Zebrafish swim into view. Nat. Rev. Genet..

[27] Nishimura Y., Murakami S., Ashikawa Y., Sasagawa S., Umemoto N., Shimada Y., Tanaka T. (2015). Zebrafish as a systems toxicology model for developmental neurotoxicity testing. Congenit. Anomalies.

[28] Lu J.L. (2010). Analysis of trends of the types of pesticide used, residues and related factors among farmers in the largest vegetable producing area in the Philippines. J. Rural Med..

[29] Chang A., Montesano M.A., Barr D., Thomas J., Geller R. (2009). Urinary elimination kinetics of acephate and its metabolite, methamidophos, in urine after acute ingestion. J. Med. Toxicol..

[30] Saera-Vila A., Kish P.E., Kahana A. (2015). Automated scalable heat shock modification for standard aquatic housing systems. Zebrafish.

[31] Kari G., Rodeck U., Dicker A.P. (2007). Zebrafish: An emerging model system for human disease and drug discovery. Clin. Pharmacol. Ther..

[32] Bernut A., Le Moigne V., Lesne T., Lutfalla G., Herrmann J.-L., Kremer L. (2014). In vivo assessment of drug efficacy against mycobacterium abscessus using the embryonic zebrafish test system. Antimicrob. Agent. Chemother..

[33] Shi X., Gu A., Ji G., Li Y., Di J., Jin J., Hu F., Long Y., Xia Y., Lu C. (2011). Developmental toxicity of cypermethrin in embryo-larval stages of zebrafish. Chemosphere.

[34] Legradi J., el Abdellaoui N., van Pomeren M., Legler J. (2015). Comparability of behavioural assays using zebrafish larvae to assess neurotoxicity. Environ. Sci. Pollut. Res..

[35] Khan T.M., Benaich N., Malone C.F., Bernardos R.L., Russell A.R., Downes G.B., Barresi M.J., Hutson L.D. (2012). Vincristine and bortezomib cause axon outgrowth and behavioral defects in larval zebrafish. J. Peripher. Nerv. Syst..

[36] Burgess H.A., Granato M. (2007). Sensorimotor gating in larval zebrafish. J. Neurosci..

[37] Stehr C.M., Linbo T.L., Incardona J.P., Scholz N.L. (2006). The developmental neurotoxicity of fipronil: Notochord degeneration and locomotor defects in zebrafish embryos and larvae. Toxicol. Sci..

[38] Mutero C.M., Ouma J.H., Agak B.K., Wanderi J.A., Copeland R.S. (1998). Malaria prevalence and use of self-protection measures against mosquitoes in Suba District, Kenya. East. Afr. Med. J..

[39] Deng J., Yu L., Liu C., Yu K., Shi X., Yeung L.W., Lam P.K., Wu R.S., Zhou B. (2009). Hexabromocyclododecane-induced developmental toxicity and apoptosis in zebrafish embryos. Aquat. Toxicol..

[40] Zeng C., Sun H., Xie P., Wang J., Zhang G., Chen N., Yan W., Li G. (2014). The role of apoptosis in MCLR-induced developmental toxicity in zebrafish embryos. Aquat. Toxicol..

[41] Yu K., Li G., Feng W., Liu L., Zhang J., Wu W., Xu L., Yan Y. (2015). Chlorpyrifos is estrogenic and alters embryonic hatching, cell proliferation and apoptosis in zebrafish. Chem.-Biol. Interact..

[42] Gu A., Shi X., Yuan C., Ji G., Zhou Y., Long Y., Song L., Wang S., Wang X. (2010). Exposure to fenvalerate causes brain impairment during zebrafish development. Toxicol. Lett..

[43] Kurps J., Broeke J.H., Cijsouw T., Kompatscher A., van Weering J.R., de Wit H. (2014). Quantitative image analysis tool to study the plasma membrane localization of proteins and cortical actin in neuroendocrine cells. J. Neurosci. Method.

[44] Casano A.M., Albert M., Peri F. (2016). Developmental apoptosis mediates entry and positioning of microglia in the zebrafish brain. Cell Rep..

[45] Livak K.J., Schmittgen T.D. (2001). Analysis of relative gene expression data using real-time quantitative PCR and the 2^−ΔΔCT^ method. Methods.

[46] Mattinen L., Kublbeck J., Rechardt O., Honkakoski P., Rautio J. (2014). Direct and rapid transcript analysis assay for CYP mRNA expression and inducibility in human primary hepatocytes. Drug Metab. Lett..

[47] Hainzl D., Cole L.M., Casida J.E. (1998). Mechanisms for selective toxicity of fipronil insecticide and its sulfone metabolite and desulfinyl photoproduct. Chem. Res. Toxicol..

[48] Brosamle C., Halpern M.E. (2002). Characterization of myelination in the developing zebrafish. Glia.

[49] Muller C., Bauer N.M., Schafer I., White R. (2013). Making myelin basic protein from mRNA transport to localized translation. Front. Cell Neurosci..

[50] Kao H.T., Porton B., Czernik A.J., Feng J., Yiu G., Haring M., Benfenati F., Greengard P. (1998). A third member of the synapsin gene family. Proc. Natl. Acad. Sci. USA.

[51] Saieva C., Aprea C., Tumino R., Masala G., Salvini S., Frasca G., Giurdanella M.C., Zanna I., Decarli A., Sciarra G. (2004). Twenty-four-hour urinary excretion of ten pesticide metabolites in healthy adults in two different areas of Italy (Florence and Ragusa). Sci. Total Environ..

[52] Weppner S., Elgethun K., Lu C., Hebert V., Yost M.G., Fenske R.A. (2006). The Washington aerial spray drift study: Children’s exposure to methamidophos in an agricultural community following fixed-wing aircraft applications. J. Expo. Sci. Environ. Epidemiol..

[53] Akoto O., Gavor S., Appah M.K., Apau J. (2015). Estimation of human health risk associated with the consumption of pesticide-contaminated vegetables from Kumasi, Ghana. Environ. Monit. Assess..

[54] Moser V.C. (2011). Functional assays for neurotoxicity testing. Toxicol. Pathol..

[55] Sano K., Isobe T., Yang J., Win-Shwe T.T., Yoshikane M., Nakayama S.F., Kawashima T., Suzuki G., Hashimoto S., Nohara K. (2016). In utero and lactational exposure to acetamiprid induces abnormalities in socio-sexual and anxiety-related behaviors of male mice. Front. Neurosci..

[56] Li H., Park G., Bae N., Kim J., Oh M.S., Yang H.O. (2015). Anti-apoptotic effect of modified chunsimyeolda-tang, a traditional Korean herbal formula, on mptp-induced neuronal cell death in a Parkinson’s disease mouse model. J. Ethnopharmacol..

[57] Clift D.E., Thorn R.J., Passarelli E.A., Kapoor M., LoPiccolo M.K., Richendrfer H.A., Colwill R.M., Creton R. (2015). Effects of embryonic cyclosporine exposures on brain development and behavior. Behav. Brain Res..

[58] Chen L., Yu K., Huang C., Yu L., Zhu B., Lam P.K., Lam J.C., Zhou B. (2012). Prenatal transfer of polybrominated diphenyl ethers (PBDES) results in developmental neurotoxicity in zebrafish larvae. Environ. Sci. Technol..

[59] Ulukaya E., Acilan C., Yilmaz Y. (2011). Apoptosis: Why and how does it occur in biology?. Cell Biochem. Funct..

[60] Spassova D., White T., Singh A.K. (2000). Acute effects of acephate and methamidophos on acetylcholinesterase activity, endocrine system and amino acid concentrations in rats. Toxicol. Pharmacol..

[61] Jung S.H., Kim S., Chung A.Y., Kim H.T., So J.H., Ryu J., Park H.C., Kim C.H. (2010). Visualization of myelination in GFP-transgenic zebrafish. Dev. Dyn..

[62] Chung A.Y., Kim P.S., Kim S., Kim E., Kim D., Jeong I., Kim H.K., Ryu J.H., Kim C.H., Choi J. (2013). Generation of demyelination models by targeted ablation of oligodendrocytes in the zebrafish CNS. Mol Cell..

[63] Jin Y., Liu Z., Peng T., Fu Z. (2015). The toxicity of chlorpyrifos on the early life stage of zebrafish: A survey on the endpoints at development, locomotor behavior, oxidative stress and immunotoxicity. Fish Shellfish Immunol..

[64] Garbarino G., Costa S., Pestarino M., Candiani S. (2014). Differential expression of synapsin genes during early zebrafish development. Neuroscience.

[65] Moser V.C. (1999). Comparison of aldicarb and methamidophos neurotoxicity at different ages in the rat: Behavioral and biochemical parameters. Toxicol. Appl. Pharmacol..

[66] Lima C.S., Dutra-Tavares A.C., Nunes F., Nunes-Freitas A.L., Ribeiro-Carvalho A., Filgueiras C.C., Manhaes A.C., Meyer A., Abreu-Villaca Y. (2013). Methamidophos exposure during the early postnatal period of mice: Immediate and late-emergent effects on the cholinergic and serotonergic systems and behavior. Toxicol. Sci..

[67] De Castro V.L., Chiorato S.H., Pinto N.F. (2000). Relevance of developmental testing of exposure to methamidophos during gestation to its toxicology evaluation. Toxicol. Lett..

