# Supplementary material for: Developmental Neurotoxicity of Methamidophos in the Embryo-Larval Stages of Zebrafish
Source: Int J Environ Res Public Health. 2016 Dec 28;14(1):23. doi: 10.3390/ijerph14010023 (PMC5295274; doi:10.3390/ijerph14010023)
Supplement: Supplementary file 1 [file ijerph-14-00023-s001.pdf]

Supplementary Materials: Developmental Neurotoxicity of Methamidophos in the Embryo-Larval Stages of Zebrafish

Xiaowei He, Jiawei Gao, Tianyu Dong, Minjian Chen, Kun Zhou, Chunxin Chang, Jia Luo, Chao Wang, Shoulin Wang, Daozhen Chen, Zuomin Zhou, Ying Tian, Yankai Xia and Xinru Wang

Table S1. Behavior observation of zebrafish larvae exposed to methamidophos at 72 hpf.

Methamidophos (μg/L)	Larvae	0	25	500
The number of escape responders	72 hpf	18.67 ± 0.67	14.00 ± 0.58 *	9.33 ± 0.88 ***

Values are expressed as mean ± SEM of three replicate samples and each assay contains 20 larvae. The number of escape responders was recorded for mechanical stimulation and the living larvae used in locomotor behavior observation all had normal morphology. * $p < 0.05$ and *** $p < 0.001$ indicate significant differences between exposure groups and the control group (Kruskal-Wallis test, with Dunn's multiple comparison test).

Table S2. The mRNA levels of *mbp* and *syn2a* in zebrafish larvae exposed to methamidophos at 72 hpf.

Methamidophos (μg/L)	0	25	500
mbp	1.00 ± 0.41	0.88 ± 0.36 *	0.57 ± 0.23 ***
syn2a	1.00 ± 0.41	0.83 ± 0.34 **	0.66 ± 0.27 ***

Data are represented as mean ± SEM of three replicate samples (20 living larvae per replicate) and are expressed as fold change relative to control. * $p < 0.05$, ** $p < 0.01$ and *** $p < 0.001$ indicate significant differences between exposure groups and the control group (Kruskal-Wallis test, with Dunn's multiple comparison test).

© 2016 by the authors; licensee MDPI, Basel, Switzerland. This article is an open access article distributed under the terms and conditions of the Creative Commons by Attribution (CC-BY) license (http://creativecommons.org/licenses/by/4.0/).
